# Toward Developing a Preventive MERS-CoV Vaccine—Report from a Workshop Organized by the Saudi Arabia Ministry of Health and the International Vaccine Institute, Riyadh, Saudi Arabia, November 14–15, 2015

**DOI:** 10.3201/eid2208.160229

**Published:** 2016-08

**Authors:** Jean-Louis Excler, Christopher J. Delvecchio, Ryan E. Wiley, Marni Williams, In-Kyu Yoon, Kayvon Modjarrad, Mohamed Boujelal, Vasee S. Moorthy, Ahmad Salah Hersi, Jerome H. Kim, on behalf of the MERS-CoV Vaccine Working Group

**Affiliations:** International Vaccine Institute, Seoul, South Korea (J.-L. Excler, I.-K. Yoon, J.H. Kim);; Shift Health, Toronto, Ontario, Canada (C.J. Delvecchio, R.E. Wiley, M. Williams);; Walter Reed Army Institute of Research, Silver Spring, Maryland, USA (K. Modjarrad);; King Abdullah International Medical Research Center, Riyadh, Saudi Arabia (M. Boujelal);; World Health Organization, Geneva, Switzerland (V.S. Moorthy);; Ministry of Health, Riyadh (A.S. Hersi)

**Keywords:** MERS-CoV, coronavirus, animal models, vaccine, prevention, therapeutic antibodies, Saudi Arabia, viruses, zoonosis, Middle East respiratory syndrome, coronavirus

## Abstract

Middle East respiratory syndrome (MERS) remains a serious international public health threat. With the goal of accelerating the development of countermeasures against MERS coronavirus (MERS-CoV), funding agencies, nongovernmental organizations, and researchers across the world assembled in Riyadh, Saudi Arabia, on November 14–15, 2015, to discuss vaccine development challenges. The meeting was spearheaded by the Saudi Ministry of Health and co-organized by the International Vaccine Institute, South Korea. Accelerating the development of a preventive vaccine requires a better understanding of MERS epidemiology, transmission, and pathogenesis in humans and animals. A combination of rodent and nonhuman primate models should be considered in evaluating and developing preventive and therapeutic vaccine candidates. Dromedary camels should be considered for the development of veterinary vaccines. Several vaccine technology platforms targeting the MERS-CoV spike protein were discussed. Mechanisms to maximize investment, provide robust data, and affect public health are urgently needed.

Middle East respiratory syndrome (MERS) remains a serious public health threat within Saudi Arabia and internationally, as recently illustrated by an outbreak in South Korea with potential pandemic risk (*1*–*7*). A vaccine (or vaccines) targeting the MERS coronavirus (MERS-CoV), which causes the disease, will be a critical component of future public health prevention measures (*8*–*10*). With the goal of accelerating the development of countermeasures against MERS-CoV, funding agencies, nongovernmental organizations, and researchers across the world assembled in Riyadh, Saudi Arabia, on November 14–15, 2015, to discuss current data and research progress to enhance understanding of disease progression from MERS-CoV infection, vaccine development, the challenges of developing treatment measures (e.g., unclear disease mechanisms and transmission patterns), preclinical development and animal models, the landscape of emerging technologies and scientific platforms, and considerations for clinical development. One primary objective of the meeting was to articulate a coordinated action plan that aligns efforts and resources. The meeting was spearheaded by the Ministry of Health (MOH) of Saudi Arabia and co-organized by the International Vaccine Institute, Seoul, South Korea.

## Development of MERS-CoV Animal Models

When developing countermeasures against MERS-CoV infection, rodents and small animal models that mimic human disease hallmarks would be useful in initial screening studies before the measure is tested in larger animals (e.g., nonhuman primates and, potentially, camels). Although upper respiratory tract disease develops more severely in the latter (*11*), studying immune correlates of protection and vaccine efficacy in camels (the only natural host besides bats and humans identified thus far) may reveal vulnerabilities of MERS-CoV that may be exploited for human vaccine strategies. 

The development of MERS vaccines faces several challenges. Existing small animal species do not naturally express the primary receptor that MERS-CoV uses to infect humans, the human dipeptidyl-peptidase 4 (hDPP4) receptor (*12*–*19*). This lack results in the animal’s inability to sustain infection and for clinical illness to develop from MERS-CoV. Larger animal models, such as nonhuman primates, have not yet been optimized to consistently mimic the disease patterns observed in human infection (which is incompletely understood) and also have associated logistical challenges because that work must be completed in Biosafety Level 3 facilities.

Mouse DDP4 cannot support MERS-CoV infection (*16*). Although efforts have been made to adapt MERS-CoV itself to exhibit human disease phenotypes in rodents, greater success has been achieved through the development of specialized mouse models that express hDPP4 (*20**–**22*). Mouse strains that globally express hDPP4 are susceptible to infection by MERS-CoV, and the mice display lower respiratory tract infection, weight loss, and increased respiratory rate, but also encephalitis, which makes the strains highly lethal. Human DPP4 expression is, however, transient and limited to the lung after Ad5-hDPP4 transduction by intranasal inoculation (*21*). These infected transgenic mice exhibit transcriptional activation of genes encoding classic antiviral cytokines (interferon [IFN]-β, IFN-γ, and MX-1) and pro-inflammatory cytokines (interleukin [IL]-2, IL-6, IL-12, p40, IL-1-α, and tumor necrosis factor [TNF]-α), as well as chemokines (granulocyte-colony stimulating factor [G-CSF], monocyte chemoattractant protein-1 [MCP-1], interferon gamma-induced protein 10 [IP-10], CXC motif ligand 1 [CXCL-1], macrophage protein 1 [MIP-1], and chemokine (C-C motif) ligand 5 [CCL5 or RANTES]), in contrast with the negligible gene activation of infected nontransgenic mice. IL-1, IL-6, TNF-α, G-CSF, MCP-1, IP-10, CXCL-1, MIP-1, RANTES, and interferon-induced GTP-binding protein (MX-1) have been detected in the lungs and brains of infected transgenic mice (*20*).

However, formation of hybrid mouse–human DPP4 dimers in transgenic mice could affect immune regulation and lead to poorly understood outcomes that could confuse the interpretation of disease natural history and vaccine efficacy. Alternatively, a minimally modified version of mouse DPP4, by mutation of 2 amino acids, can support MERS-CoV infection. Mice with this mutation experience severe lower respiratory tract infection, although they do not exhibit brain infection (*16*). In addition, Pascal et al. have developed mice that express hDPP4, under the control of its endogenous promoter and the 3′ untranslated region, and show lung-specific infection and inflammation (*22*). Further testing may prove that vaccine evaluation in these small animal models could lead to a better understanding of immunogenicity and efficacy of vaccine candidates and the therapeutic measures being considered for evaluation in larger animals and humans.

Among current nonhuman primate models, rhesus macaques display mild-to-moderate clinical signs on viral challenge (*23*), whereas the common marmoset is reported to exhibit more severe signs of infection (*24*,*25*) and could be a better model for the severe clinical syndrome observed in MERS-CoV–infected persons. However, not all research groups have been able to replicate severe disease outcomes in marmosets. Factors contributing to this could include variations in physical location, age, and origin of the marmosets; challenge virus strains and stocks; route and dose of inoculation; and in protocols. To be able to provide robust and reproducible outcomes, these nonhuman primate models need additional development, optimization, and standardization. 

Camels are also being used to evaluate MERS-CoV infection, and findings from Adney et al. showed that these animals are unique in that they experience an upper respiratory tract infection. Although we do not know the efficiency of airborne versus droplet or another mode of transmission, viral shedding from the upper respiratory tract might explain the efficiency of camel–camel and camel–human transmission (*11*,*26*). Although camels do not display the severe disease symptoms observed in infected humans (*26*), a camel infection model remains useful for understanding the disease in camels and identifying potential immune correlates of protection induced by vaccination. Veterinary countermeasures could form part of a One Health strategy to forestall zoonotic transmission to humans (*1*). Hesitation to implement animal vaccination strategies in camels once a vaccine becomes available can be attributed to the absence of severe disease in camels (only upper respiratory tract infection with rhinitis) and to skepticism among key groups regarding zoonotic transmission of MERS-CoV to humans (e.g., camel breeders).

Although the animal models for evaluating MERS-CoV infection represent progress, they do not recapitulate the pathogenesis of severe human disease. A combination of both small and large animal models should be considered for evaluation of preventive and therapeutic candidates for MERS. Regardless of the chosen model, comparing and interpreting results effectively and reducing discrepancies among laboratories will be crucial for researchers to agree on a set of standards with respect to experimental design, including variables for age of animals, specimen handling, route of administration, type of virus challenge, inoculation schedule, sample collection, and disease scoring algorithms.

## Pipeline of MERS-CoV Vaccine and Antibody Technologies

Building on the experience from the closely related severe acute respiratory syndrome coronavirus (SARS-CoV) (*27*), researchers have been actively working to understand MERS-CoV genetics to inform vaccine and therapeutic development efforts. They quickly demonstrated that the spike (S) protein, a viral surface glycoprotein, was essential for recognition of hDPP4 and viral entry into cells and likely represented a prime target for immunogen design for the development of vaccines and monoclonal antibodies (*18*,*28*,*29*). At the workshop, we reviewed various approaches—all in preclinical development and all based on the S protein or one of its components, including nanoparticles, subunit proteins and peptides, DNA, various viral vectors, and live attenuated MERS-CoV.

Nanoparticles formed with MERS-CoV S protein, under development by Novavax (Gaithersburg, Maryland, USA), have been shown to induce virus neutralizing antibodies (NAbs) in mice after a single injection; proprietary adjuvants enhance this response (*30*). Vaccines using antigens expressed from the baculovirus platform developed by Novavax have been evaluated in human subjects in the context of phase I and phase II clinical studies for other infectious diseases without notable vaccine-related safety concerns (*31*–*33*).

Portions of the S protein, specifically the receptor-binding domain (*8*,*29*,*34*–*36*), are also being developed as subunit vaccines. Jiang noted that these fragments map to a “critical neutralizing region” and induce strong immune responses and NAbs in mouse models (*37*,*38*). Moreover, the subunit vaccines have been shown to protect transgenic mice when challenged with MERS-CoV, indicating that vaccines focused on the receptor-binding domain may be sufficient for protective immunity to develop against the virus (*39*,*40*).

Several viral vectors, including adenovirus (*41*,*42*), modified vaccinia Ankara (MVA) (*43*,*44*), and measles virus (*45*), are also under development by different groups. Various lengths of S protein are being expressed on these platforms and are able to generate antibodies in animals that can neutralize MERS-CoV in vitro and, at least for some vector platforms, also generate cellular immune responses (*43*,*45*). For MVA- and measles virus–based vaccines, these responses confer protection in hDPP4-expressing mice (*43*,*45*). MVA constructs, which have established safety profiles in humans, have been tested in camels and can induce protective immunity, representing a potential veterinary technology (*46*). Moreover, on the basis of supportive data from animal studies, these MVA constructs will soon enter clinical trials. Vaccines based on live attenuated viruses historically have been shown to be highly efficacious; they are also safe and generally well tolerated. Enjuanes and others reported to the group the development of 2 engineered MERS-CoV vaccine candidates. One candidate was based on a propagation-defective MERS-CoV strain, and another was a live attenuated virus with 3 safety guards that used a MERS-CoV infectious cDNA clone (*47*). An inactivated SARS-CoV vaccine was shown to be safe and able to induce NAbs in a phase I trial (*48*).

DNA vaccines are generally perceived as a safe, stable platform for in vivo antigen expression. A SARS-CoV DNA vaccine, which expresses the SARS-CoV S protein, has been shown to induce NAbs and functional T-cell responses in humans (*49*). GeneOne (Blue Bell, PA, USA) is developing a proprietary, full-length S protein DNA vaccine candidate that has been shown to induce NAbs and highly functional T cells in various animal models and protect rhesus macaques from infection after MERS-CoV challenge when the vaccine is administered with electroporation to enhance uptake of the plasmid DNA (*50*). Concerns remain regarding the immunogenicity of DNA vaccines in humans, although the effects of using therapeutic vaccination strategies for other diseases raise the potential for DNA-only approaches (*51*). In addition, Modjarrad reported that using a prime-boost format, a full-length S protein DNA vaccine, followed by an S-protein boost, can increase NAb titers, reduce the clinical severity of MERS, and increase the durability of protection in macaques (*52*).

To complement active immunization approaches, researchers are also advancing several prophylactic or therapeutic approaches against MERS-CoV using NAb technologies through preclinical development. Because these NAbs target epitopes of the S protein (or specifically the receptor-binding domain), they can cause precise and potent inhibitory effects on viral entry in small and large animal models (*53*). The mechanisms of neutralization have been uncovered and are typically mediated by blocking MERS-CoV binding to hDPP4 (*22*,*52*,*54*–*56*). As the supplementary agents of antibodies, the peptidic MERS-CoV fusion inhibitors targeting the conserved region in the S protein HR1 domain region are highly potent in inhibiting infection of MERS-CoV strains, including those resistant to NAbs. Intranasal administration of the peptides protected hDPP4-transgenic mice from MERS-CoV challenge, suggesting that, alone or in combination with NAbs, these peptides could be used to prevent and treat MERS-CoV infection (*37*,*39*,*57*).

Further characterization of these technologies and the potential for combination approaches are ongoing as investigators tackle questions related to viral escape (*58*,*59*). Preliminary results indicate that viruses that evade antibody neutralization have reduced viral fitness, demonstrating that escape can occur but comes at a cost to fitness. Nevertheless, continued investigation and surveillance are warranted. Marasco noted that sequencing of circulating strains will be critical to monitor viral evolution (*60*), which will only be possible with increased sample- and data-sharing. Ongoing studies related to cross-reactivity with human tissue and the effects of polyclonal and nonneutralizing antibodies are also underway as passive immunotherapy becomes more accepted to prevent and treat MERS-CoV infection.

Overall, selecting specific technologies and approaches that warrant further development is difficult, given the diversity of models and readouts and the concomitant need for greater standardization in the field. Although each technology presents unique advantages and deficiencies related to desired immunogenicity, safety, durability of protection, need for adjuvant, and manufacturing considerations, some technologies have a long track record in the clinic, which would potentially simplify their development and regulatory pathway. Given the public health urgency, these platforms (or combinations thereof) should be made a priority.

The experience with SARS-CoV offers a sobering lesson: countermeasures that advance on the basis of promising preclinical data may ultimately exacerbate disease in humans. Antibody-dependent enhancement of infectivity has been observed in cell culture in which a human promonocyte cell line is used *61*–*63*). In mice and hamsters vaccinated with a recombinant native full-length SARS-CoV S protein trimer, serum IgG developed that blocked binding of the S protein to the ACE2 receptor and neutralized SARS-CoV infection in vitro. SARS-CoV entered human B-cell lines in an FcγRII-dependent and ACE2-independent fashion, indicating that antibody-dependent enhancement of virus entry is a novel cell entry mechanism of SARS-CoV. Vaccinated animals showed no signs of enhanced lung pathology or hepatitis, and viral load was undetectable or greatly reduced in lungs after challenge with SARS-CoV (*64*). However, in the presence or absence of adjuvant, vaccination of mice with viruslike particles or inactivated virus induced eosinophilic immunopathologic changes in young and aged mice (*65*–*67*). The pulmonary immunopathologic features, on challenge with SARS-CoV, were associated with Th2-type immunopathology with prominent eosinophil infiltration. Although no enhancement of immunopathologic features has been observed in MERS-CoV–vaccinated and –challenged animals, future studies of MERS-CoV vaccines in animals and humans should consider that possibility.

## Vaccine Development Considerations for MERS-CoV

To date, commitment to open communication regarding MERS-CoV vaccine development has been haphazard, and leaders in the field are calling for a new approach that integrates resources to accelerate science and enhance biosecurity. New norms and standards are under development by the World Health Organization (WHO) to streamline sample collection (type, storage and availability, quality control); and information dissemination and publication. Combining resources available in Saudi Arabia, South Korea, the United States, Europe, and beyond to develop countermeasures for MERS-CoV, an “open innovation” paradigm shift can maximize public sector investment, provide robust information for a systems-level approach, and deliver the necessary public health effect that is urgently needed.

The Saudi Arabia MOH, working with Saudi academic institutions, WHO, and other stakeholders, recognizes the crucial role it has to play in defining the public health goals that will guide vaccine development efforts for MERS-CoV (*68*,*69*). Researchers, vaccine developers and health authorities must understand how a vaccine is expected to fit into the larger public health strategy to combat MERS (e.g., target populations and vaccination strategies, level of efficacy, safety profile for a vaccine), as well as the pathway to future vaccine testing (e.g., design of efficacy trial), licensure and access. Few vaccine developers in the MERS arena have experience conducting preclinical and clinical research in the Middle East, and the Saudi MOH and Saudi Food and Drug Authority have a valuable role to play in defining the expectations for future clinical studies and in educating developers on the associated regulatory pathway.

## Summary

The potential threat posed by MERS-CoV necessitates a multipronged approach to the development of effective countermeasures. Salient public health messages from the workshop included the following points:

1. Accelerating the development of a vaccine requires a better understanding of MERS-CoV epidemiology, transmission, and pathogenesis in humans and animals. This information will help develop target product profiles for human and veterinary vaccines, which in turn will facilitate planning for efficacy trials and inform development strategies.

2. Because current animal models do not fully reflect hallmarks of severe human disease, a combination of both rodents and nonhuman primate models should be considered in evaluating and developing preventive and therapeutic candidates under standardized conditions.

3. Current vaccine development strategies involve a variety of technology platforms, primarily targeting the MERS-CoV S protein. Given the public health urgency, platforms (or combinations thereof) with an established safety track record in humans should be given priority. Other target species such as dromedary camels should also be considered for the development of veterinary vaccines as a One Health approach.

4. Attention should be paid to lessons learned from SARS-CoV vaccine development efforts, particularly to signs of potential disease enhancement in various animal models.

5. Therapeutic antibodies are recognized as potentially useful tools in MERS prevention and treatment, but the concern around escape mutants with increased fitness, although concern is not limited to this technology type, warrants continued investigation and surveillance. Such an approach could be considered alone or in combination with vaccine approaches. As supplementary agents, the peptidic fusion inhibitors may be developed as MERS prophylactics and therapeutics.

6. An opportunity exists for greater coordination around specific technology platforms and to ensure that appropriate incentives are considered to stimulate research and development collaboration from academia, industry, nongovernmental organizations, and governments.

7. The Saudi Arabia MOH, working with WHO and other stakeholders, has a crucial role to play in defining the public health goals that will guide vaccine development efforts.

## Next Steps: Establishing a New Paradigm for Collaboration

Funding agencies, nongovernmental organizations, and companies recognize the need for cooperation and have resolved to formalize a collaborative model. The field recognizes the opportunity to set a precedent for how it collaborates as a global community in the context of an emerging disease, building on lessons learned from the recent international response to the Ebola epidemic. Although still subject to consultation, key components of a partnership(s) were identified, including coordinating funding, sharing samples/data, advancing preclinical models, beginning clinical trials in regions having outbreaks, and standardizing assays and reagents for testing.

Exact partnership structures remain to be determined but should at the very least allow for coordination of activities through frequent, transparent, and open discussions among funding agencies and stakeholders. Future models, including a formalized consortium of players who would make a long-term commitment to advance selected products through development phases, can be contemplated once technologies are evaluated more rigorously. Regardless of the final partnership structure(s), the core of any collaborative strategy should include sharing of data and samples and standardizing laboratory assays to ensure that everyone learns from each other, are able to compare technologies, and ultimately accelerate the development of new solutions.
